# Massive Pulmonary Embolism Presenting as Recurrent Seizures: Successful Thrombolysis and Thrombectomy Guided by Clinical Suspicion

**DOI:** 10.7759/cureus.104644

**Published:** 2026-03-04

**Authors:** Cyril Busschots Martins, Jonathan De Bodt

**Affiliations:** 1 Department of Emergency Medicine, Hôpital de Mons - Site Kennedy, CHU HELORA, Mons, BEL

**Keywords:** arterial catheter, cardiac arrest, clinical suspicion, low-flow state, mechanical thrombectomy, obstructive shock, point-of-care ultrasound, pulmonary embolism, seizures, thrombolytic therapy

## Abstract

Massive pulmonary embolism (PE) with obstructive shock is a life-threatening condition associated with poor prognosis, primarily due to diagnostic challenges. Clinical presentation is often nonspecific, beyond the classic signs of shock, and confounding factors, such as seizures secondary to cerebral hypoperfusion, as observed in this case, may further obscure the diagnosis. We report the case of a 54-year-old male patient brought to the ED by the mobile emergency and resuscitation service following recurrent seizures associated with refractory hypotension. Given the rapid clinical deterioration and multiple cardiac arrests, an underlying cause was urgently sought. After ruling out common etiologies such as sepsis and acute coronary syndrome, massive PE was strongly suspected based on bedside point-of-care echocardiography findings, elevated D-dimer levels, and high clinical suspicion. Systemic thrombolysis (alteplase) was administered directly in the ED, leading to hemodynamic stabilization. The diagnosis was later confirmed via contrast-enhanced thoracic CT, and the patient subsequently underwent mechanical thrombectomy, resulting in complete recovery. He was extubated and regained full consciousness on the same day. This case illustrates how timely management guided by strong clinical suspicion can lead to full recovery without sequelae. It also emphasizes the critical importance of minimizing no-flow time to achieve optimal outcomes.

## Introduction

Pulmonary embolism (PE) is a common condition, with an estimated annual incidence of 45 cases per 100,000 patients [1-3], and it accounts for approximately 5-10% of in-hospital cardiac arrests (IHCAs) [4,5]. Survival after cardiac arrest due to obstructive shock in high-risk PE remains critically low, with mortality rates reaching up to 90% in some studies [4-7]. Prognosis is even worse when the presentation is atypical, observed in 3% of cases [3], or when cardiac arrest occurs within two hours of symptom onset [5,6]. The case we report herein exhibits both of these high-risk features simultaneously, as seizures occur in only 1-2% of PE cases [1].

Despite extensive study, PE presents with nonspecific and heterogeneous clinical manifestations [1,3,4]. Although CT pulmonary angiography (CTPA) is the gold standard for definitive diagnosis, its use may be limited by the patient’s hemodynamic instability [4]. In the setting of hemodynamic compromise, particularly during cardiac arrest, identifying PE is exceedingly challenging. Consequently, the decision to administer thrombolytic therapy often relies solely on indirect evidence, and the risk-benefit balance must be carefully weighed, given the potential for secondary bleeding. This diagnostic and therapeutic uncertainty likely explains why only few randomized controlled trials have evaluated thrombolysis during cardiac arrest secondary to PE [6,7].

## Case presentation

A 54-year-old male patient with a history of behavioral disorders and epilepsy (secondary to cerebral anoxia following a cardiac arrest from a road traffic accident in 2011) was brought to the ED by the mobile emergency and resuscitation service for recurrent seizures (three episodes lasting less than five minutes over a 40-minute period) and hypotension (82/52 mmHg) without tachycardia (90 bpm).

After initial management with 500 mL of NaCl 0.9% and 10 mg of intravenous diazepam, the patient remained conscious but slightly somnolent. However, hypotension proved refractory and did not improve despite a second 500 mL fluid bolus. Concurrently, progressive desaturation developed, and the first arterial blood gas (ABG) analysis revealed hypoxemia and hypocapnia with mild respiratory alkalosis but no lactic acidosis (pH: 7.50; PaCO₂: 27 mmHg; PaO₂: 61 mmHg). Despite improvement in respiratory parameters with high-flow nasal oxygen (Optiflow^®^), the patient’s condition rapidly deteriorated, with worsening hypotension, loss of consciousness, clinical signs of shock, and ultimately cardiac arrest.

This cascade of events challenged the initial diagnosis of status epilepticus and raised suspicion of an underlying condition causing cerebral hypoperfusion in the context of pre-existing brain injury.

Advanced life support (ALS) was immediately initiated according to the European Resuscitation Council guidelines. The initial rhythm was pulseless electrical activity (PEA). Epinephrine (1 mg) was administered every four minutes, and early intubation was performed. No-flow time was zero minutes, as the arrest occurred in the presence of medical personnel. Return of spontaneous circulation (ROSC) was achieved after seven minutes.

The admission ECG was unremarkable, and an infectious etiology was initially suspected, supported by elevated CRP (263 mg/L; reference range: <5 mg/L). Empiric treatment with amikacin (25 mg/kg) and hydrocortisone (100 mg) was initiated. Despite insertion of a central venous catheter and arterial line and continuous norepinephrine infusion at 0.5 µg/kg/min, the patient remained hemodynamically unstable and experienced three additional cardiac arrests within the following hour. Each arrest was promptly managed with chest compressions and 1 mg boluses of epinephrine, with ROSC achieved in under four minutes each time.

Between two cardiac arrests, bedside echocardiography was performed, revealing marked right ventricular dilation at the expense of the left ventricle, which appeared hyperdynamic. The diagnosis of obstructive shock thus became highly probable, and D-dimer testing was urgently added to the laboratory workup on suspicion of massive pulmonary embolism. D-dimers were markedly elevated at 41.7 mg/L (reference range: <0.5 mg/L). Meanwhile, ABG demonstrated severe metabolic acidosis with lactic acidosis but no hypoxemia under mechanical ventilation (pH: 7.10; PaCO₂: 42 mmHg; PaO₂: 434 mmHg).

Given the patient’s critical condition, thrombolytic therapy with alteplase (Actilyse^®^) at 0.6 mg/kg over 15 minutes was administered following the fourth cardiac arrest. Approximately five minutes after thrombolysis, the patient experienced a fifth and final cardiac arrest, which was successfully reversed after two minutes of chest compressions and 1 mg of epinephrine.

**Figure 1:**
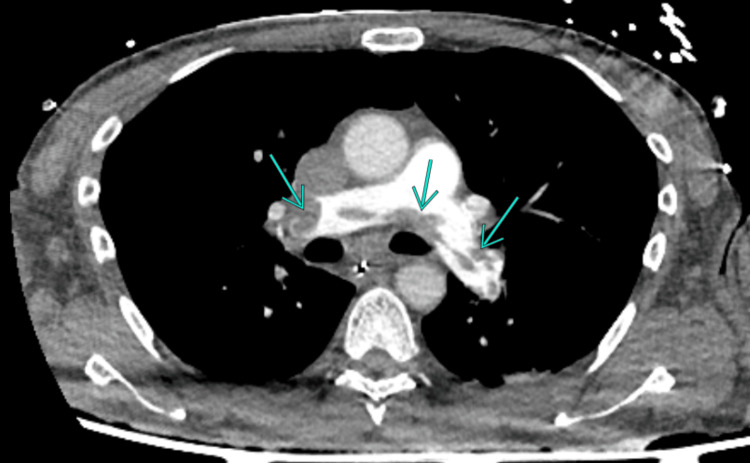
CTPA showing bilateral filling defects consistent with thrombi in the pulmonary arteries CTPA, CT pulmonary angiography

**Figure 2:**
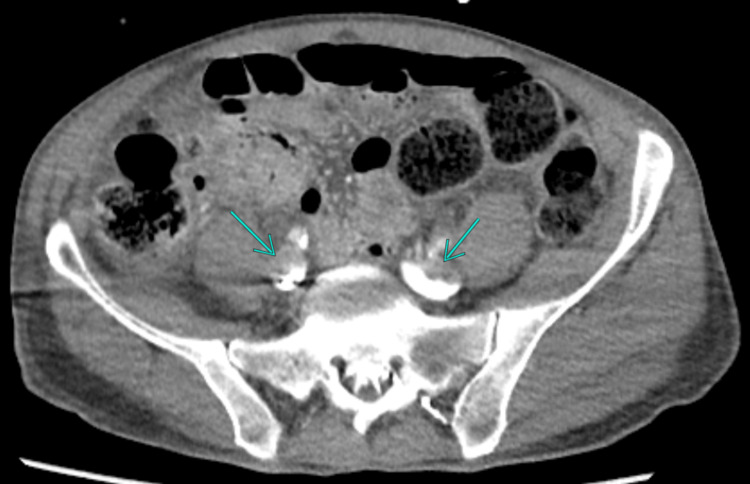
Portal-phase CT angiography of the abdomen demonstrating venous thrombi in the common iliac veins

The patient subsequently stabilized, allowing transfer to the CT scanner, which confirmed bilateral massive PE (Figure 1) with cardiac repercussions, likely secondary to a large thrombus extending from the iliac veins to the inferior vena cava (Figure 2).

**Figure 3:**
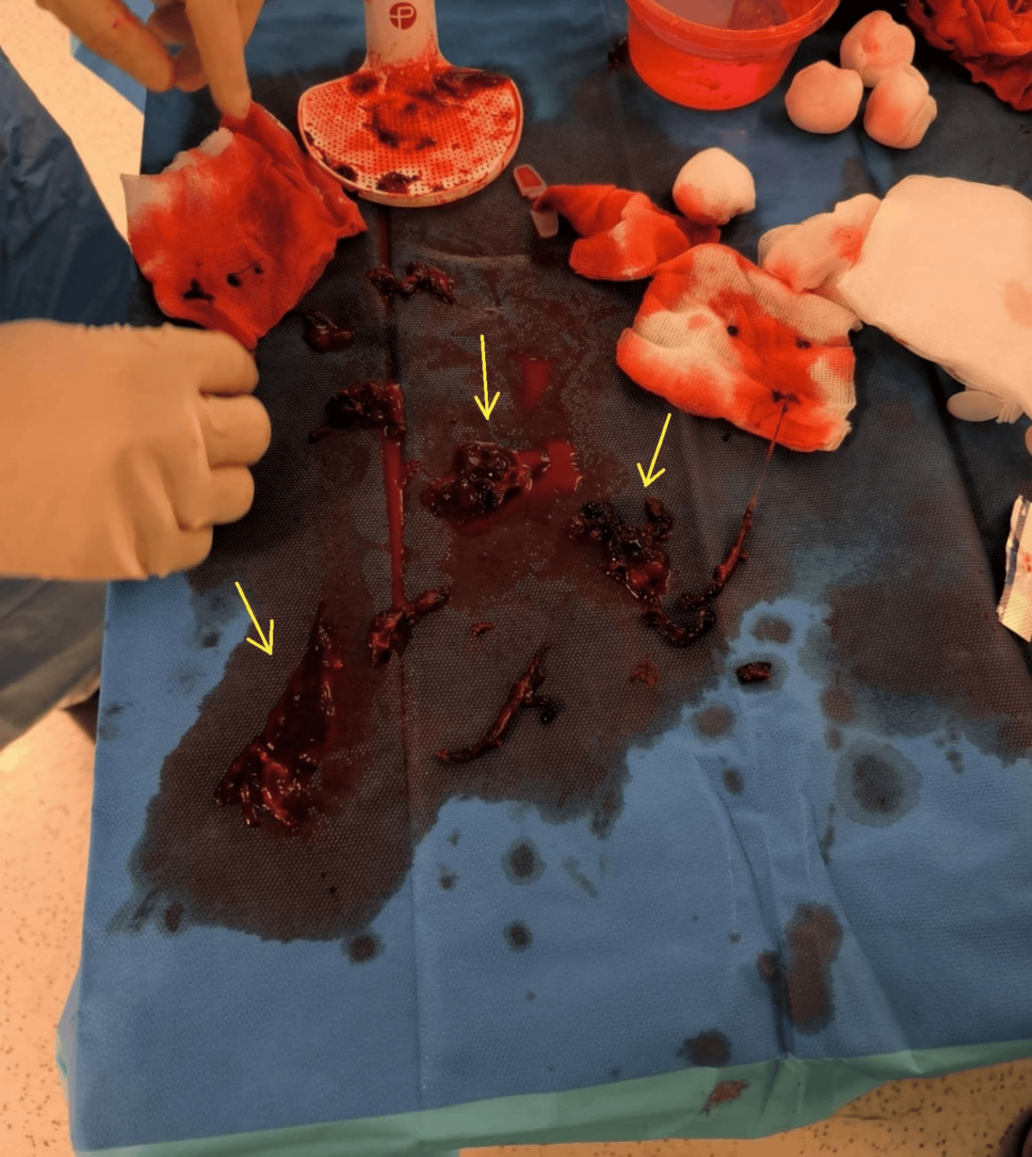
Gross pathology specimen showing thromboemboli retrieved from the pulmonary arteries

In the afternoon, interventional radiologists performed mechanical thrombectomy, successfully retrieving large thrombi from the pulmonary arteries (Figure 3). The patient was subsequently admitted to the ICU. His clinical condition improved rapidly, and he was extubated the same evening, regaining full consciousness and being weaned off all vasopressor support.

An electroencephalogram performed the following day showed no epileptiform activity, indicating an excellent functional prognosis at 24 hours, despite an initially estimated high-risk PESI score of 174. The patient was transferred to the pulmonary ward after four days in the ICU and was discharged home 10 days later without any sequelae. He was prescribed dabigatran (Pradaxa^®^) for anticoagulation.

## Discussion

While PE is a well-characterized condition, its emergency diagnosis remains challenging due to nonspecific or heterogeneous clinical presentations [1,5-7] and the reliance on CTPA for confirmation, which requires hemodynamic stability [4]. PE accounts for approximately 10% of out-of-hospital cardiac arrests (OHCAs) [7,8] and 5-10% of IHCAs [4,6].

In massive PE, the initial presentation may manifest as obstructive shock. This type of shock is particularly difficult to identify and manage due to its low etiological proportion (1-4% of all shock cases) and the limited efficacy of supportive measures. Clinical improvement is unlikely until the obstructive cause, such as tension pneumothorax, cardiac tamponade, or massive PE, is resolved. Given the rapid deterioration of these patients, extracorporeal membrane oxygenation (ECMO/extracorporeal life support) may occasionally be required [6,8,9].

Thrombolysis is currently the recommended treatment for PE with refractory obstructive shock (hemodynamic instability). Recent studies, including PEITHO-3, suggest favorable outcomes, though these trials have involved small cohorts [4,6,9,10]. The increased risk of intracranial hemorrhage (~2%) limits its use [4,10], necessitating careful case-by-case assessment of the risk-benefit ratio. Importantly, when thrombolysis is administered, resuscitation efforts should be extended up to 90 minutes post-administration, with improved outcomes reported in some studies [4,5,9].

Mechanical thrombectomy can be performed as an adjunct to fibrinolytic therapy. Recent trials, such as FLASH [11] and FLARE [12], suggest improved survival and quality of life [10-12]. However, few studies have addressed peri-arrest management of PE, given methodological and ethical challenges [4,6,10]. Surgical embolectomy is another alternative but was not pursued in this case [13].

Despite these therapeutic options, diagnosis remains difficult, particularly in peri-arrest settings where clinical signs may be absent. Pre-arrest signs may suggest obstructive shock, but history and contextual factors become critical for diagnosis once cardiac arrest occurs, as clinical manifestations often disappear. Some studies have attempted to identify peri-arrest diagnostic clues, such as the predominance of PEA, observed in 63% of cases (versus 32% for asystole and 5% for ventricular fibrillation [6]). However, these findings have proven inconclusive for establishing a reliable diagnosis.

As with any cardiac arrest, systematic evaluation of reversible causes using the “4Hs and 4Ts” approach is recommended [6,7]. In our case, this framework helped exclude alternative diagnoses, while transient ROSC allowed bedside investigations and initiation of thrombolytic therapy based on strong clinical suspicion.

Standard ALS protocols emphasize the ABC(DE) sequence, which is primarily symptom-oriented, providing a uniform, teachable framework for healthcare providers (physicians, nurses, and paramedics). However, this approach can oversimplify the search for reversible causes (4Hs and 4Ts). A deeper understanding of shock pathophysiology and less common etiologies should be incorporated into training. Point-of-care ultrasound (POCUS), arterial catheterization, and targeted laboratory tests (e.g., ABGs and D-dimers) should play a central role in the management of critically ill patients, provided they do not interfere with resuscitation maneuvers [4-6].

In this case, the patient’s initial seizures could have led to misdiagnosis, as systemic hypoperfusion likely reactivated preexisting cerebral sequelae, triggering the seizures. The markedly elevated CRP was likely due to a systemic inflammatory response from massive PE rather than infection. This highlights the importance of not being misled by atypical presentations and underscores that any signs of shock should prompt thorough investigation while minimizing no-flow time in the event of deterioration or cardiac arrest.

In such cases, advanced monitoring tools are essential to guide management. Beyond POCUS, early arterial catheter placement in highly unstable patients, either just before cardiac arrest or immediately after ROSC, can be invaluable. Cerebral hypoperfusion often develops before electrocardiographic changes, and noninvasive blood pressure monitoring (cuff-based) is unreliable in rapidly deteriorating patients, particularly in prehospital settings [14]. Neurological alterations are early indicators of hypoperfusion but become uninterpretable once the patient is intubated. According to recent guidelines from the French Society of Emergency Medicine and the French Society of Anesthesia and Intensive Care, a mean arterial pressure of at least 65 mmHg (or 60 mmHg in permissive hypotension) is recommended to ensure adequate cerebral perfusion [15,16].

Currently, PEA is an indication for resuscitation and is grouped with asystole in ALS algorithms. However, PEA encompasses a broad clinical spectrum. A patient in shock with undetectable blood pressure (sinus rhythm on ECG but unmeasurable cuff pressure) still meets the PEA definition and should receive resuscitative efforts. Yet, is the prognosis the same as for electrocardiographic asystole? Unlike asystole, the type of PEA described above represents an early stage of cardiac arrest, suggesting that distinguishing these two pathophysiological entities could help tailor management strategies. This underscores the potential benefit of arterial catheterization, which allows for the early detection of impending arrest, enabling earlier initiation of resuscitation and reduced no-flow time.

Arterial catheterization enables early detection of impending arrest, facilitating earlier resuscitation and reduced no-flow time. In trained hands, femoral arterial catheter placement takes only a few minutes (7 ± 2 minutes, according to studies) [14,17,18] and should be considered in the peri-arrest setting, with or without Doppler ultrasound guidance [19]. This procedure does not delay resuscitation, as it can be performed concurrently with other interventions, and it offers substantial benefits for management optimization and neurological outcomes.

Prehospital arterial catheterization in highly unstable patients may also be feasible. With prehospital ECMO already under investigation, femoral arterial catheterization, well within the scope of emergency physicians, could significantly improve OHCA outcomes, particularly during long transport times or when external monitoring is unreliable, such as in moving vehicles [17]. Further prehospital studies are needed, but this approach could demonstrate improved survival and quality of life, complementing the established in-hospital benefits [16-18]. Invasive blood pressure monitoring in the prehospital setting could prove as beneficial as end-tidal CO₂ monitoring while serving as a complementary tool.

Despite the generally poor prognosis associated with cardiac arrest due to obstructive shock, our patient recovered fully and in a remarkably short time. We attribute this favorable outcome primarily to the early recognition of hypoperfusion and the minimization of no-flow time, emphasizing their critical role in patient prognosis. Accordingly, we advocate for the strong consideration of arterial catheterization in peri-arrest management, both in hospital and prehospital settings.

## Conclusions

PE and the resulting obstructive shock remain among the most lethal causes of cardiac arrest. In this case, the patient’s remarkable recovery without sequelae, despite a PESI score of 174 and five cardiac arrests, was achieved through minimization of no-flow time, facilitated by early arterial catheterization for hypoperfusion detection, and targeted therapy with thrombolysis followed by mechanical thrombectomy. This highlights how rapid shock classification and etiology-specific interventions can substantially improve outcomes in peri-arrest settings, where every minute counts.

Accordingly, advanced hemodynamic monitoring, including arterial catheterization and POCUS, should be considered essential competencies for emergency physicians managing unstable patients, particularly those with ROSC. While standard ALS protocols provide a foundational framework, their limitations in distinguishing shock subtypes underscore the need for supplemental tools to guide time-sensitive decisions. Future studies should assess the feasibility and impact of prehospital arterial catheterization, which may be particularly valuable in settings with prolonged transport times or unreliable noninvasive monitoring.

## References

[1] Lam M, Jammal M, Tiev K (2012). Pulmonary embolism revealed by a seizure: a case report and literature review [Article in French]. Rev Med Interne.

[2] Motte S (2006). Diagnostic and therapeutic management of pulmonary embolism [Article in French]. Rev Med Brux.

[3] Grégoire G, Devalet B (2024). Abdominal pain as a presenting symptom of pulmonary embolism [Article in French]. Ann Fr Med Urgence.

[4] Bartha C, Milos RI, Yerna M (2019). Cardiac arrest due to pulmonary embolism successfully treated with systemic thrombolysis: management and outcome [Article in French]. Louvain Med.

[5] Kürkciyan I, Meron G, Sterz F (2000). Pulmonary embolism as a cause of cardiac arrest: presentation and outcome. Arch Intern Med.

[6] Laher AE, Richards G (2018). Cardiac arrest due to pulmonary embolism. Indian Heart J.

[7] Welle SR, Harrison MF (2021). Massive pulmonary embolism causing cardiac arrest managed with systemic thrombolytic therapy: a case report. Am J Case Rep.

[8] Pudil J, Rob D, Smalcova J (2023). Pulmonary embolism-related refractory out-of-hospital cardiac arrest and extracorporeal cardiopulmonary resuscitation: Prague OHCA study post hoc analysis. Eur Heart J Acute Cardiovasc Care.

[9] Jansen A, Schmitt CJ, Cabrera D, Wieruszewski ED (2025). Retrospective review of thrombolytic use for cardiac arrest due to suspected pulmonary embolism. Am J Emerg Med.

[10] Bengueddache-Schweblin C, Zbinden S, Roffi M, Righini M, Glauser F (2024). Mechanical thrombectomy in pulmonary embolism [Article in French]. Rev Med Suisse.

[11] Khandhar S, Jaber W, Bunte MC (2023). Longer-term outcomes following mechanical thrombectomy for intermediate- and high-risk pulmonary embolism: 6-month FLASH Registry results. J Soc Cardiovasc Angiogr Interv.

[12] Tu T, Toma C, Tapson VF (2019). A prospective, single-arm, multicenter trial of catheter-directed mechanical thrombectomy for intermediate-risk acute pulmonary embolism: the FLARE Study. JACC Cardiovasc Interv.

[13] Akhlaghpasand M, Mohammadi I, Hajnorouzali A (2025). Salvage pulmonary embolectomy following cardiac arrest: a 10-year experience. Ann Med Surg (Lond).

[14] Hasegawa D, Sato R, Duggal A, Schleicher M, Nishida K, Khanna AK, Dugar S (2024). Comparison of central and peripheral arterial blood pressure gradients in critically ill patients: a systematic review and meta-analysis. Crit Care Explor.

[15] Lamontagne F, Richards-Belle A, Thomas K (2020). Effect of reduced exposure to vasopressors on 90-day mortality in older critically ill patients with vasodilatory hypotension: a randomized clinical trial. JAMA.

[16] Drumheller BC, Pinizzotto J, Overberger RC, Sabolick EE (2021). Goal-directed cardiopulmonary resuscitation for refractory out-of-hospital cardiac arrest in the emergency department: a feasibility study. Resusc Plus.

[17] Aziz S, Barratt J, Starr Z, Lachowycz K, Major R, Barnard EB, Rees P (2024). The association between intra-arrest arterial blood pressure and return of spontaneous circulation in out-of-hospital cardiac arrest. Resuscitation.

[18] Onishi H, Matsuyama T, Yasutake Y (2023). Arterial and venous pressure monitoring during cardiopulmonary resuscitation for out-of-hospital arrests: four case reports. J Vasc Dis.

[19] Cohen AL, Li T, Becker LB (2022). Femoral artery Doppler ultrasound is more accurate than manual palpation for pulse detection in cardiac arrest. Resuscitation.

